# A video-based analysis of situations bearing the risk of respiratory disease transmission during football matches

**DOI:** 10.1038/s41598-022-07121-7

**Published:** 2022-02-22

**Authors:** Oliver Faude, Simon Müller, Sebastian Schreiber, Jonas Müller, Lukas Nebiker, Florian Beaudouin, Tim Meyer, Florian Egger

**Affiliations:** 1 Department of Sport, Exercise and Health, University of Basel, Basel, Switzerland; 2 Institute of Sport and Preventive Medicine, Saarland University, Saarbrücken, Germany

**Keywords:** Infectious diseases, Disease prevention

## Abstract

We aimed to analyze the number and type of contacts involving the risk of respiratory disease transmission during football match play. We analysed 50 matches from different playing levels. Two reviewers evaluated the contacts of all players in each match. We focused on between-player contacts, crowding, actions with potentially increased aerosol and droplet production and within-player hand-to-head contacts. We categorized the duels with direct contact into frontal and other ones and measured contact duration. The number of between-player contacts were similar between playing levels (median 28.3 [IQR 22.6, 33] contacts per player-hour). Frontal contacts summed up to 8% of all contacts. Contacts involving the head occurred less than once per player and match with none lasting longer than 3 s. Crowding included between two and six players and the duration was mostly less than 10 s. Aerosol and droplet producing activities were three to four times more frequent in adult compared to youth players. Our results suggest that the risk of respiratory pathogen transmission is low during football matches. This conclusion is based on the finding that most close contact situations are of short duration and on the fact that it is an outdoor sport.

## Introduction

Football (association football, soccer) is a high-intensity sport including some contacts between players. During a situation like the COVID-19 pandemic, it has become highly relevant to know whether the execution of team sports including football is associated with an increased risk of the transmission of respiratory pathogens. Currently it is unknown, whether the number and nature of physical contacts is of such a character that the risk of respiratory disease transmission is increased while playing football. Transmission of respiratory pathogens mainly occurs via three different pathways: (i) through pathogen-containing respiratory droplets (e.g. coughing, sneezing, talking), (ii) through airborne transmission of infectious aerosols, and (iii) through contact infection (e.g. touching mucous membranes with contaminated body parts or objects)^1–3^.

The scientific literature on infectious diseases in team sports is currently limited. An early literature review on infectious diseases in rugby players focused mainly on blood-borne viruses such as HIV or hepatitis B and C^4^. Another review article on infectious disease outbreaks in competitive sports reporting the most common route of transmission in outbreaks was direct human-to-human contact, while airborne and vector-borne transmissions were rare^5^. This refers mostly to Herpes simplex virus and methicillin-resistant *Staphylococcus aureus* (MRSA) infections. Similarly, Collins et al.^6^ found that infectious disease outbreaks mainly occurred in competitive sports with close contacts and involved the skin and soft tissue with MRSA being the most predominant pathogen. Valid information on the risk of disease by respiratory transmission of pathogens is currently scarce^7^.

Within the last year, few studies have been published which assessed the risk of SARS-CoV-2 transmission in team sports. Jones, Phillips, Kemp, Payne, Hart, Cross and Stokes^8^ analysed four rugby league matches in England in which eight infected players participated. The authors conducted video and GPS analyses and followed all players during the period after the matches with RT-PCR testing and symptom monitoring. Though five new infections occurred after the matches, the authors concluded based on the type of contacts during match play and detailed analyses of potential alternative ways of transmission that transmission risk on the pitch is negligible. Egger, Faude, Schreiber, Gärtner and Meyer^9^ conducted a very similar study in football and observed no transmission during a two-week period after three matches with 18 infected participating players in total. In a more comprehensive study, analyzing 104 matches and training sessions in amateur, youth and professional football with 165 potentially infectious SARS-CoV-2 positive players on-field transmission risk appeared to be low^10^. Video-analysis of 21 matches with 34 potentially infectious players in the same study revealed that football-specific contacts were likely not sufficient to transmit the virus. In order to estimate the potential infection risk of players, Randers, Knudsen, Thomasen, Panduro, Larsen, Mohr, Milanovic, Krustrup and Andersen^11^ analysed GPS data in youth (8 and 9 years old) and adult (20–33 years old) recreational players with regard to the time and number of close contacts (within a 1.5 m zone) during different small-sided game formats (3 vs. 3 to 8 vs. 8). The average duration of a close contact was slightly more than 1 s. The authors concluded that during small-sided football the time spent within potentially dangerous zones is brief. Similarly, Goncalves, Mendes, Folgado, Figueiredo, Travassos, Barros, Campos-Fernandes, Beckert and Brito^12^ applied dynamic tracking analysis of one elite football match in order to evaluate the feasibility of this approach to estimate interpersonal contacts within 2 m zones. These authors also found only short interpersonal contact times while playing football.

The purpose of the present study was to go beyond existing papers and analyze more specifically situations, which potentially bear a risk for respiratory transmission of pathogens during football matches. We examined all transmission-relevant physical contacts during football matches, both between players and within a player. We additionally aimed at evaluating the risk of contagion on different levels of play (youth, amateur, professional), if there is a difference between matches played during spring compared to fall and if the introduction of specific behavioural measures after the first COVID-19 lockdown in spring 2020 affected the occurrence of such contacts in the German Bundesliga.

## Methods

We analysed a total of 50 football matches. Matches were chosen based on different criteria: (i) the level of play (professional vs. amateur (5th German league) vs. youth, i.e. under-11 and under-13 age groups), (ii) the time of the year during which the matches were played (spring vs. fall), (iii) the availability of video recordings of appropriate quality (uncut video recordings; standard view at the level of midline), and, particularly regarding professional football, (iv) whether the matches were played before or after the first German lockdown (i.e. after the introduction of specific measures to minimise transmission risk).

Twenty professional matches were provided by the German Football League (Deutsche Fußball-Liga, DFL). We chose randomly five matches from May 2019, five matches from November 2019 and ten matches from May 2020 (after the first COVID-19 lockdown and subsequent implementation of hygiene measures). Amateur matches were freely available on commercial webservers (youtube.com, sporttotal.tv) and were analysed similar to the professional matches at two different times of the year (five matches from May 2019 and five matches from November 2019). With regard to youth football, we analysed 20 matches (due to the lower playing time and number of players in under-11 and under-13 age group football, 50 to 60 min per match, seven to nine players per team). We chose ten matches from April and May 2018 and ten matches from October and November 2018. Youth videos were recorded as part of the UEFA Heading Study^13^. For each match, we documented the time of day the match took place as well as the weather conditions and the actual temperature based on freely available online data (agrarwetter.net, timeanddate.de). The study protocol is in accordance with the guidelines of the Declaration of Helsinki and was approved by the local ethics committee (Ärztekammer des Saarlandes, Ethikkommission; proposal number 21/17).

Two independent reviewers performed the video analysis of each single match. They evaluated the quantity and quality of contacts of all players as well as the referee in the field of view during the matches. In total, four reviewers performed the analyses, of which two reviewers were a priori randomly assigned to each match. Before analyzing the first match, all reviewers were instructed with regard to potentially risky situations and behaviour. Reviewers together analysed various 10 min samples of random football matches, in total about 90 min. Thereby, reviewers a priori differentiated and elaborated the definitions of potentially risky actions. All reviewers as well as the principal investigators approved the final categorization system (Table 1), which we then applied for all video analyses. During the main analysis, the reviewers and the investigators were able to exchange views via a discussion platform, so that in individual cases contentious video scenes could always be shared.

**Table 1:** Definitions of risk situations and risk behavior during football matches.

Situation or behavior	Specification
Between-player physical contacts (duels) (as contacts per player-hour)	Total number of contacts excluding contacts to the lower limbs (duration and direction, frontal (face-to-face, i.e. collisions, scramble in anticipation of a corner kick/free-kick) or other direction)
	Upper body contacts: shoulder–shoulder, arm–arm, front–back, hand–jersey, hand–hand, hugging
	Head contacts: head–head, arm-/hand-to-head
Crowding during breaks (as contacts per playing-hour)	Gatherings of at least two persons (players or referee) within a range of less than about 1.5 m
	Number of involved players and duration
	In free-kick walls
	While celebrating goals
	During breaks before corners/free-kicks
	During injury breaks
Aerosol and droplet producing activity (as number per player-hour)	Speaking or shouting (within a range of less than about 1.5 m), spitting
Within-player hand-to-head contacts (as contacts per player-hour)	Touching the own head with the hand, with or without touching a mucous membrane (mouth, nose, eyes)
Hand-/head-to-ball contacts (as contacts per playing-hour)	Before set-play: throw-in, corner, free-kick, goal-kick, kick-off, drop ball
	During match play: kick-out, goalkeeper save (block or catch), header

We focused on between-player contacts (duels, i.e. upper body contacts) and behaviour increasing the risk of aerosol and droplet infection. Based on their orientation we categorized the duels with direct contact into frontal (face-to-face) and other ones and measured the duration of each contact. We further evaluated the number of players and the duration when crowding within a range of about 1.5 m occurred. We counted within-player hand-to-head contacts (e.g. touching the mouth or hair, i.e. with or without contact to a mucous membrane) and all hand-to-ball contacts during match play, such as throw-ins, kick-outs and attempts to place the ball before corner-kicks, free-kicks, goal kicks and kick-offs, catches and blocks of the goalkeeper. All videos were analysed in real time and stopped or rewound as often as needed to capture all relevant contacts and behaviours of each player. When the number of observed situations differed between the reviewers, we took the higher number for statistical analysis in order to arrive at a conservative estimate of risk situations. We calculated intraclass correlation coefficients (ICC) in order to assess the inter-observer agreement. ICCs ranged from 0.34 to 0.94 with 74% being moderate to excellent (ICC > 0.5)^14^. Inter-individual shoulder- and arm-contacts, direct head-to-head contacts and intra-individual hand-to-head contacts should be interpreted with caution (ICC < 0.5).

### Statistical analysis

We present all parameters mainly descriptive as medians with interquartile range (IQR). Additionally, we related inter-individual and intra-individual contacts and behaviour to playing exposure as incidents per player-hour. We calculated match exposure by multiplying the mean number of visible persons (players plus referee) with playing time (90 min for adults matches, 60 min for under-13 matches, 50 min for under-11 matches; not considering the actual playing time including extra time and or a reduced number of players due to a red card). For this purpose, all reviewers counted the number of visible persons at six time points during each match (three time points while playing and three time points during breaks each evenly distributed over the playing time). All set-play situations were normalized to playing time and data are given as incidents per playing-hour.

We applied the Kruskal–Wallis rank test to assess potential differences between matches played in spring vs. fall, for professional matches played before vs. after the COVID-19 lockdown in the German Bundesliga as well as to analyze potential differences between playing levels (professional vs. amateur vs. youth). We interpret the P values as a continuous measure of compatibility of the data with the statistical model and not relative to an arbitrary significance threshold^15^. In addition, we calculated median differences between the different categories with bootstrapped 95% confidence intervals based on 5000 resamples^16^. For statistical analyses, we used Jamovi software as well as the freely accessible website http://www.estimationstats.com.

## Results

The average number of visible persons (players plus referee) on the pitch was 12.6 in professionals (55% of total; 18.9 player-hours per match), 13.1 in amateur male players (57% of total; 19.7 player-hours per match) and 10.4 in youth matches (58% of total; 10.4 player-hours per match). Temperature ranged from 11 to 26 °C (mean with standard deviation: 16.9 (3.4) °C) during spring matches and from 0 to 21 °C (8.8 (5.8) °C) during fall matches. Weather conditions were sunny (27% of all matches in spring, 10% in fall), mixed (7% in spring, 15% in fall), cloudy (67% in spring, 65% in fall) or rainy (0% in spring, 10% in fall).

### Between-player contacts (duels)

Between-player contacts were similar between pre- and post-lockdown in professional football as well as between spring and fall matches (see Supplementary Material Table S1). The only exception were hand-to-hand contacts, which were more frequent in fall matches compared to spring and pre-lockdown compared to post-lockdown.

The overall number of between-player contacts was similar between playing levels with an average of nearly one contact every 2 min. The number of contacts lasting longer than 3 s was about twice as high in amateur players compared to professional and youth players (Table 2). Frontal contacts summed up to eight percent of all contacts between players. We found similar numbers of upper body contacts (duels) between playing levels. Contacts involving the head occurred on average less than once per player during a 90-min match. Head contacts differed between playing levels with amateur players showing about one contact per player-hour more than professional and youth players. The same is true for both sub-categories head-to-head and arm-to-head contacts. We observed no frontal head contacts and no head contacts lasting longer than 3 s.

**Table 2:** Between-player contacts representing potential risk situations associated with the transmission of respiratory diseases. Data for the different playing levels are presented as frequency per player-hour, as absolute numbers (contacts of more than 3 s duration) and as median with interquartile range. Differences between playing levels are displayed as median differences with 95% confidence intervals. P values from Kruskal–Wallis test.

	All matches	Professional	Amateur	Youth	Δ Youth–professional	Δ Amateur–professional	Δ Amateur–youth	P value
**All contacts**	28.3 (22.6, 33)	27.1 (24.2, 31.8)	30 (24.8, 31.7)	26.6 (20.6, 33.6)	− 0.4 (− 7.6, 7.1)	2.9 (− 3.2, 8.2)	3.3 (− 4.9, 9.7)	0.74
> 3 s	9 (6, 16)	9 (6.8, 11.5)	18.5 (14, 25.3)	7.5 (5.8, 9.3)	− 1.5 (− 4.5, 1)	9.5 (2, 17)	11 (3.5, 18)	0.005
**Upper body contacts**	27.3 (22.1, 32.8)	26.2 (23.9, 31.5)	27.9 (24.2, 30.4)	26.5 (20.6, 33.4)	0.3 (− 7.3, 7.8)	1.7 (− 4.5, 5.9)	1.4 (− 6.6, 8.2)	0.84
Shoulder–shoulder	4.2 (3.2, 4.9)	3.8 (3, 4.3)	4.2 (3.5, 6)	4.4 (3.5, 5)	0.6 (− 0.6, 1.7)	0.4 (− 0.8, 2.3)	− 0.3 (− 1.5, 1.9)	0.18
Arm–arm	7.6 (5.8, 9.4)	7.8 (6.6, 9.6)	6.6 (5.5, 7.3)	8.2 (5.5, 10.7)	0.4 (− 2.3, 2.7)	− 1.2 (− 2.7, 0.3)	− 1.6 (− 3.9, 1.1)	0.14
Front–back	2.7 (2, 3.4)	2.9 (2.4, 3.3)	3.4 (3.1, 3.8)	1.9 (1.6, 2.4)	− 1 (− 1.5, − 0.5)	0.5 (− 0.1, 1.1)	1.5 (0.8, 2.1)	< 0.001
Hand–jersey	10.1 (8.4, 13.4)	10 (9, 14.9)	11.4 (9.5, 11.5)	9.8 (7.9, 13.9)	− 0.3 (− 4.4, 3.5)	1.4 (− 1.1, 2.7)	1.6 (− 1.3, 3.8)	0.81
Hand–hand	1.3 (0.7, 2.2)	1.2 (0.7, 2.3)	2 (1.2, 2.2)	1.3 (0.7, 1.7)	0.1 (− 0.8, 0.7)	0.8 (− 0.2, 1.6)	0.7 (− 0.3, 1.4)	0.35
Hugging	0	0 (0, 0.1)	0 (0, 0.1)	0	0 (− 0.1, 0)	0 (− 0.1, 0.1)	0 (0, 0)	0.003
**Head contacts**	0.3 (0.1, 0.5)	0.3 (0.3, 0.5)	1.3 (0.5, 2.0)	0 (0, 0.2)	− 0.3 (− 0.6, − 0.3)	1 (0.1, 2)	1.3 (0.4, 2.3)	< 0.001
> 3 s	0	0	0	0				–
Head–head	0	0 (0, 0.1)	0.3 (0.1, 0.6)	0	0 (− 0.1, 0)	0.3 (0, 0.6)	0.3 (0.1, 0.5)	< 0.001
Arm–head	0.3 (0.1, 0.5)	0.3 (0.2, 0.4)	1.0 (0.4, 1.3)	0	− 0.3 (− 0.3, − 0.3)	0.6 (0.1, 1.4)	1 (0.4, 1.6)	< 0.001

### Crowding during breaks

The number of players involved in crowding and the duration of crowding were similar in spring compared to fall matches as well as in professional matches played before and after the lockdown, except for goal celebrations (see Supplementary Material Table S2). After the lockdown, only one third of players was involved in crowding after a goal was scored and the duration of goal celebrations was considerably shorter (from about 10 s down to less than 2 s).

Crowding included on average between two to three (free-kick walls) and six players (prior to corners and free-kicks) with goal celebrations and injury breaks lying in between (Table 3). The duration of crowding was in most instances less than 10 s, except for free-kick walls and injury breaks in adult matches.

**Table 3:** Number of involved players and duration of crowding during breaks. Data for the different playing levels are presented as frequency per match-hour and as median with interquartile range. Differences between playing levels are displayed as medians with 95% confidence intervals. P values from Kruskal–Wallis test.

	All matches	Professional	Amateur	Youth	Δ Youth–professional	Δ Amateur–professional	Δ Amateur–youth	P value
**Free-kick wall**								
N players	2.5 (2, 3)	2.2 (2, 3)	2.5 (2.5, 3.1)	2.4 (0, 2.9)	0.3 (− 0.7, 0.9)	0.4 (− 0.3, 1)	0.1 (− 0.4, 1.3)	0.38
Duration (s)	15 (8.4, 21.7)	21.4 (13.8, 24.8)	18.6 (16.3, 22)	8.9 (0, 14.8)	− 12.6 (− 22.3, − 5.5)	− 2.9 (− 8.9, 3.9)	9.8 (3.3, 18.6)	< 0.001
**Goal celebration**								
N players	4.3 (0, 6.4)	4.7 (2.5, 6.5)	6.7 (6.1, 8.8)	0 (0, 3.3)	− 4.7 (− 6.4, − 2.1)	2 (− 0.1, 4.5)	6.7 (4.8, 9)	< 0.001
Duration (s)	4 (0, 8.9)	4.7 (1.3, 11.3)	9.1 (6.3, 15.5)	0 (0, 4)	− 4.7 (− 11.1, − 1.5)	4.4 (− 1.3, 11.8)	9.1 (5.5, 16.8)	0.001
**Corner/free-kick**								
N players	5.7 (4.6, 6.5)	6.2 (5.6, 7.6)	6.7 (5.8, 7.3)	4.3 (4, 5)	− 1.9 (− 3.2, − 1.2)	0.5 (− 0.8, 1.5)	2.4 (1.2, 3.3)	< 0.001
Duration (s)	6.5 (5.1, 8.4)	7 (5.8, 8.1)	11.4 (8.5, 16.3)	5.2 (3.8, 6.5)	− 1.8 (− 3.6, 0.3)	4.4 (0.9, 10.8)	6.2 (2.6, 12.5)	< 0.001
**Injury**								
N players	4.6 (3.6, 5.9)	4.2 (3.4, 4.8)	5.5 (4.3, 7.9)	5.1 (0, 6)	0.9 (− 0.3, 2.3)	1.4 (− 0.2, 4.1)	0.4 (− 1.4, 3.5)	0.09
Duration (s)	9.9 (5.6, 15)	10.4 (6.2, 14.9)	13.8 (10.2, 27.6)	7 (0, 10.8)	− 3.4 (− 8.5, 1)	3.5 (− 3.7, 18.2)	6.9 (0.8, 22)	0.04

### Actions of individual players with the potential risk of increased aerosol and droplet transmission

Aerosol and droplet producing activities, like speaking, shouting and spitting, were similar in matches played in spring compared to fall. Such activities, particularly speaking, showed lower values in post-lockdown matches (see Supplementary Material Table S3).

**Figure 1:**
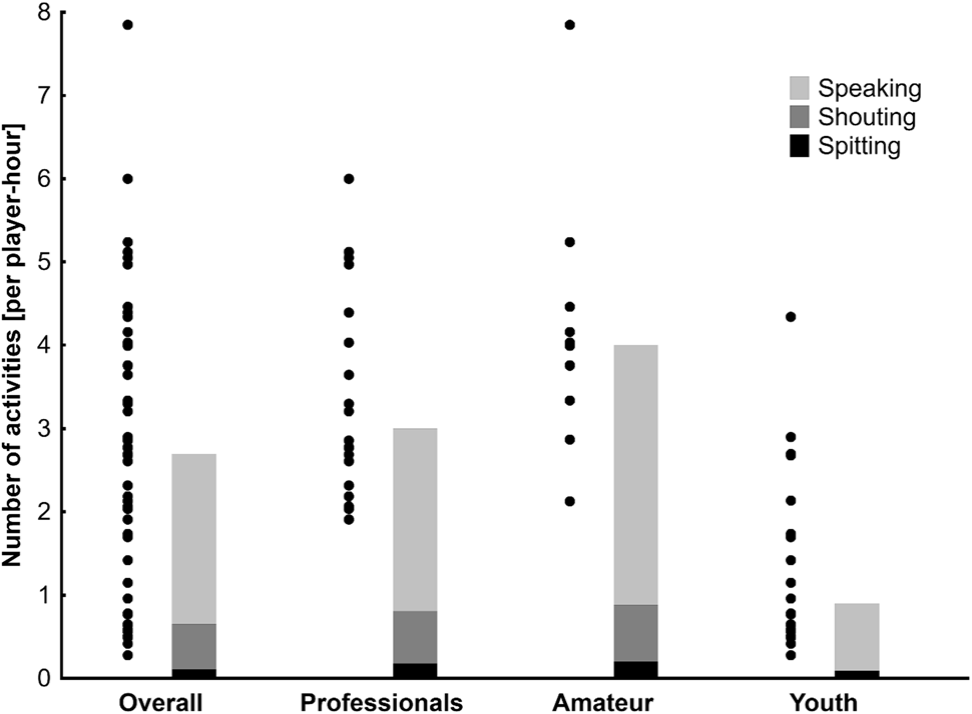
Actions of individual players representing potential risk situations associated with the transmission of respiratory diseases (by aerosol or droplet production). Individual data points are mean values for each match (N = 20 professional, N = 10 amateur, N = 20 youth). The bars represents the median of all activities partitioned into the sub-categories speaking, shouting and spitting.

Speaking within a distance of 1.5 m of another player summed up to about three quarters, shouting to 4% and spitting to 20% of those activities, respectively. Such aerosol and droplet producing activities were more frequent in adult players (professionals median 3 [IQR 2.5, 4.2]; amateurs: median 4 [IQR 3.3, 4.5]) compared to youth players (median 0.9 [IQR 0.6, 1.7], P < 0.001; Fig. 1). In professional players the frequency was 2.2 [IQR 1.3, 3.4] and in amateur players 3.1 [IQR 2.3, 4.1] per player-hour higher.

### Within-player hand-to-head contacts

Within-player hand-to-head contacts were similar in matches played during spring vs. fall as well as in professional matches played before and after the lockdown (see Supplementary Material Table S3).

We observed differences in within-player hand-to-head contacts between the different playing levels with professional players showing the lowest values and youth players the highest total number of contacts and non-mucosal contacts, while amateur players showed the highest values for mucosal contacts (Table 4). Seventy-six percent of all within-player hand-to-head contacts occurred during breaks.

**Table 4:** Within-player hand-to-head contacts during match play. Data are presented as frequency per player-hour and as median with interquartile range. Differences between playing levels are displayed as median differences with 95% confidence intervals. P values from Kruskal–Wallis test.

	All matches	Professional	Amateur	Youth	Δ Youth–professional	Δ Amateur–professional	Δ Amateur–youth	P value
**Hand-to-head**	22.2 (16.9, 29.6)	16.7 (15, 18.8)	26.5 (24, 29.6)	29.2 (21.3, 38.3)	12.5 (5.5, 19.7)	9.8 (6.5, 13.7)	− 2.7 (− 10.9, 4.5)	< 0.001
Mucosal	12.4 (10.1, 16)	10.9 (10, 11.8)	16.5 (16, 17.1)	13.3 (9.6, 15.3)	2.4 (− 1.1, 4.2)	5.6 (3.9, 6.8)	3.2 (0.7, 6.5)	0.002
Non-mucosal	9.6 (6.3, 13.9)	6 (4.6, 6.6)	11.4 (9.3, 12.4)	16.8 (10.7, 22.6)	10.8 (6.3, 15.5)	5.5 (3, 7.1)	− 5.4 (− 10.2, − 0.7)	< 0.001

### Hand-/head-to-ball contacts before set-play and during match play

All hand- or head-to-ball contacts were similar in spring compared to fall matches as well as in professional matches played before and after the lockdown (see Supplementary Material Table S4).

Hand-to-ball contacts before throw-ins, goal-kicks, corners, kick-outs and kick-offs and due to goalkeeper saves were two- to fourfold more frequent in youth compared to professional players with amateurs lying in between. Hand-to-ball contacts before free-kicks and headers, however, were two to three times more frequent in amateur compared to youth matches with professionals lying in between (Table S5).

## Discussion

Our observational study on situations, which potentially bear the risk of transmission of respiratory pathogens, revealed that between-player contacts occur on average every 2 min with contacts to the head, frontal contacts or contacts lasting longer than 3 s being very rare. In most instances, crowding of two to six players lasted no longer than 10 s. Aerosol and droplet producing activities and direct contacts to mucous membranes are infrequent in football matches. The time of the year as well as the first lockdown seem to have only minor effects on such situations. Differences in playing level are present and may affect infection risk.

Direct close contact between players enabling droplet transmission remains the most likely way of on-pitch transmission^1,17^. This can occur during direct contact with an infected player, with face-to-face contacts being the most risky ones^2^. Such contacts, however, were rare in our study, occurring on average every 2 min for an individual player with the majority of contacts not being in direct frontal positioning. On average, a single player had one frontal contact with an opponent about every 18 min during the match. Most contacts were arm-to-arm, shoulder-to-shoulder or contacts between the hand and the opponents’ jersey during duels. Noteworthy, contacts involving the head, which are particularly relevant for infection, occurred on average less than once per player and match. Transmission risk is also a direct function of contact duration^17^. In our study, nearly all contacts lasted for less than 3 s. This finding is in line with results of Randers, Knudsen, Thomasen, Panduro, Larsen, Mohr, Milanovic, Krustrup and Andersen^11^ who analysed the time amateur players spend within a radius of 1.5 m to another player. Approximately 90% of contacts within this risk zone were shorter than 3 s, and less than 0.5% of all contacts lasted longer than 10 s. The average contact time was slightly above 1 s. Similarly, Goncalves, Mendes, Folgado, Figueiredo, Travassos, Barros, Campos-Fernandes, Beckert and Brito^12^ analysed a professional soccer match by means of a tracking system. The average accumulated time within a radius of two meter for a given player-player combination was 32 s per match. In summary, these findings show that players move only very briefly within or through risk zones. For purposes of risk assessment for respiratory infectious disease transmission, football should therefore be considered a sport with brief, sporadic contacts between players instead of the classic categorization as “contact sport”.

Crowding involved on average two to six players with the highest player numbers prior to corners and free-kicks. Such situations, where players prepare for an optimal position to score or to defend a goal, can be regarded particularly hazardous as players are staying close together and struggling for a superior positioning compared to the direct opponent. Crowding, however, in the majority of cases lasted less than 10 s and, therefore, the risk of transmission of a pathogen might be considered low, particularly in an open-air setting^17,18^. Interestingly, the number of players involved and the duration of crowding during goal celebrations was considerably reduced after the lockdown in the German Bundesliga. This might have been due to increased awareness and strict guidelines outside the pitch based on the current hygiene measures. Recent data from the following season, however, show that both, the values for the number of players and the duration of crowds during goal celebration, have turned back to the values observed before the lockdown, i.e. this was only a very short-term effect (unpublished own data).

Aerosol and droplet producing activities like speaking, shouting and spitting represent situations, which bear a particular risk of pathogen transmission via small particles^1,2^. Such activities occurred about four times per match for each player and, thus, may be considered being less relevant from an infection perspective. This is particularly true with regard to potential SARS-CoV-2 infection as the probability of aerosol transmission in an outdoor environment is very low^3,17–19^. Interestingly, professional players spoke considerably less with each other within a short distance (about 1.5 m) during the re-start period after the lockdown compared to before. One may speculate that this behaviour change was due to an increased awareness of aerosols and droplets potentially playing a role in virus transmission (or at least of situations with potential increased submission risk).

Contacts by a single player with his hand to the head bear the risk of direct transfer of a pathogen to mucous membranes if this player had contact with respiratory secretions of an infected player^2^, either directly or via a contaminated surface, e.g. the ball, an opponents’ arm or jersey. Such contacts within an individual player occurred on average about every 3 min. In slightly more than half of those cases a mucous membrane (eyes, nose, mouth) was touched by the player. More than three quarters of these contacts happened during breaks. These numbers are similar to data reported for men during simulated train rides^20^ or for medical students during lectures^21^. Thus, it can be carefully argued that face touching, a potential vector for transmission of respiratory viruses, occurred in a similar frequency as if players were in other daily life situations. Consequently, playing football does not lead to an increased risk for such routes of transmission.

Potential hand-to-ball contacts by the goalkeeper, during breaks before set-play or head-to-ball contacts while heading may also (marginally) contribute to an increased transmission risk due to direct contamination with the contagion (contact route). Our data show the frequency of such situations during football match play, too. We cannot conclude on the actual risk of transmission in these situations. However, it has been suggested that contact with contaminated objects, though possible, plays a minor role with regard to transmission^3,22^. Furthermore, the ball represents an object with a continuously moving surface, which is constantly rubbed off and thus probably represents a smaller source of infection compared to stationary surfaces. Interestingly, we found large differences in the frequency of hand-to-ball contacts between playing levels. We can only speculate about potential reasons. Situations like throw-ins, corners and goal kicks occurred more frequently in youth matches. This observation might be due to the smaller fields in youth football and potentially worse technical skills, leading to more situations where the ball is played out of the field. Even though we cannot conclude on the exact reasons for these discrepancies, our observations may inform stakeholders about potential differences between playing levels and slightly differing targeted measures to reduce infection risks for these particular groups.

Current evidence suggests that main risk factors for the transmission of SARS-CoV-2 (and respiratory pathogens with similar transmission routes) are close contacts while crowding in a confined environment with the duration of the contact being an important modifier^17^. Our results on the nature of contacts during football match play suggest that playing football bears merely a low risk of infection on the pitch. This finding is in line with the studies by Jones, Phillips, Kemp, Payne, Hart, Cross and Stokes^8^ as well as by Egger, Faude, Schreiber, Gärtner and Meyer^9^ who were not able to identify an infection on the pitch, though in total 26 players participated during four rugby and three football matches. These findings were confirmed by a larger study analyzing more than 100 matches or training sessions in which 165 infected players participated^10^. The authors were able to exclude on-field transmission in all but one case. Similarly, two studies evaluated the return-to-sport process in summer 2020 in the United States. Drezner, Drezner, Magner and Ayala^23^ reported two out of 1906 youth players being tested positive during a 6-week period, both infections could be ruled out to have occurred during training. Watson, Haraldsdottir, Biese, Goodavish, Stevens and McGuine^24^ observed 282 positive cases (in youth players and club staff) during a 73 days follow-up period when restarting recreational football in more than 90,000 players during more than 45,000 training sessions and more than 6000 matches. Only one infection was finally considered to be attributed to playing football. Furthermore, the incidence rate in these youth players was considerably lower than in the general population and independent of the training being conducted with or without contacts. Comparable findings showing that on-field infections are very unlikely to occur or that the incidence or prevalence in football players is not higher than that of the general population have been published for professional football in Germany^25,26^, Denmark^27^ and Qatar^28^. In summary, current evidence suggests a limited risk of transmission of respiratory pathogens while playing outdoor team sports.

### Methodological considerations

A particular strength of our study is that we evaluated youth, amateur and professional football, i.e. different levels of play, which facilitates generalizability of our findings. We analysed a large sample of matches during different times of the year and before and after the lockdown, which was associated with general recommendations regarding behaviour change in potentially risky situations from the perspective of infection prevention. All evaluations regarding the distance between players (while crowding, speaking or shouting) were done visually by two reviewers and, thus, should be considered rough estimates and not exact values. Inter-observer agreement was heterogeneous for the different obtained parameters and, thus, should be interpreted cautiously in some instances. However, for most parameters agreement between reviewers was sufficient. Assessment can be objectified by means of dynamic tracking data as shown by Goncalves, Mendes, Folgado, Figueiredo, Travassos, Barros, Campos-Fernandes, Beckert and Brito^12^. From our data, we cannot directly conclude on real virus transmission on the pitch. Thus, our findings should merely be regarded as indirect assessment of transmission risk. Further, we only analysed players and the referee, who were actively involved in match play. Conclusions with regard to transmission risk in other areas, settings and circumstances associated with playing football (e. g. dressing rooms and team meetings) cannot be drawn.

## Perspective

Our data provide information about potential risk situations for the transmission of respiratory pathogens while playing football. Though we designed and conducted this study in consequence to the COVID-19 pandemic, these data potentially can be applied to other respiratory infectious agents, too. We found that the risk of pathogen transmission, in general, is likely low in most situations with football being an outdoor sport with only limited contact. These data, particularly the differences regarding playing level, can be used by all stakeholders in order to introduce targeted measures to reduce the risk of respiratory disease transmission while playing football.

## Supplementary Information

Supplementary Information 1.

Supplementary Information 2.

Supplementary Tables.

## Data Availability

The data set used for the statistical analyses have been made freely available at https://osf.io/uzxny/ as well as in the Supplementary Material. Additionally, a list with the match details and the corresponding web links of the amateur matches is provided. The professional matches have been made available by the German Football League (DFL), which is the copyright holder. In case of specific requests with clear scientific interest, we can have a release checked by the DFL in each individual case.
