# Supplementary material for: A video-based analysis of situations bearing the risk of respiratory disease transmission during football matches
Source: Sci Rep. 2022 Feb 22;12:3034. doi: 10.1038/s41598-022-07121-7 (PMC8863802; doi:10.1038/s41598-022-07121-7)
Supplement: Supplementary file 1 — Supplementary Information 1. [file 41598_2022_7121_MOESM1_ESM.docx]

10 Spiele der Fußball Oberliga Rheinland-Pfalz/Saar

**Sommer 2019**

Spiel 37: Wiesbach – Koblenz (10.05.2019)

https://sporttotal.tv/mavBwa4QD

Spiel 38: Mechtersheim – Kaiserslautern 2 (17.05.2019)

https://sporttotal.tv/maQArle30

Spiel 39: Trier – Emmelshausen (18.05.2019)

https://www.youtube.com/watch?v=9i55vF1nD34

Spiel 40: Emmelshausen – Koblenz (22.05.2019)

https://sporttotal.tv/ma79QaGV1

Spiel 41: Wiesbach – Dilligen (22.05.2019)

https://sporttotal.tv/madAXnNYv

**Winter 2019**

Spiel 42: Engers– Koblenz (31.10.2019)

https://sporttotal.tv/mamvw0WKw

Spiel 43: Koblenz Pfeddersheim (09.11.2019)

https://www.youtube.com/watch?v=OD1iAWUwoOQ

Spiel 44: Trier – Mainz (09.11.2019)

https://www.youtube.com/watch?v=qKgNCz85t_E

Spiel 45: Koblenz – Wiesbach (23.11.2019)

https://www.youtube.com/watch?v=-VCAp-vyEeQ

Spiel 46: Diefflen – Koblenz (30.11.2019)

https://www.youtube.com/watch?v=sCqOC5w1Pq4
