# Supplementary material for: A video-based analysis of situations bearing the risk of respiratory disease transmission during football matches
Source: Sci Rep. 2022 Feb 22;12:3034. doi: 10.1038/s41598-022-07121-7 (PMC8863802; doi:10.1038/s41598-022-07121-7)
Supplement: Supplementary file 3 — Supplementary Tables. [file 41598_2022_7121_MOESM3_ESM.docx]

**TABLE S1** Between-player contacts representing potential risk situations associated with the transmission of respiratory diseases. Data are presented as frequency per player-hour and as median with interquartile range. Differences between matches played in spring vs. fall and before and after the lockdown are displayed as median differences with 95% confidence intervals. P values from Kruskal-Wallis test.

|  | **spring** | **fall** | **Δ** | **P value** | **pre lockdown** | **post lockdown** | **Δ** | **P value** |
| --- | --- | --- | --- | --- | --- | --- | --- | --- |
| All contacts | 29.3 (23.7,34.8) | 27.7 (21.8,31.9) | -1.5 (-8,4.5) | 0.33 | 25.9 (24.6,28.5) | 30.3 (24.3,38.1) | 4.4 (-3.4,14.4) | 0.23 |
| Upper body contacts | 28.2 (23.4,34.5) | 26.9 (21.5,30.9) | -1.3 (-7.4,4.6) | 0.24 | 24.8 (24.2,27.8) | 30.1 (23.9,37.6) | 5.3 (-2.5,16.7) | 0.20 |
| Shoulder-shoulder | 4.3 (3.4,4.9) | 3.5 (3.1,5) | -0.8 (-1.4,0.2) | 0.41 | 3.1 (2.8,4) | 4.2 (3.8,4.5) | 1.1 (-0.1,2) | 0.07 |
| Arm-arm | 7.8 (6.5,10.4) | 6.8 (5.5,8.7) | -1.1 (-2.8,0.8) | 0.10 | 7.8 (6.5,8.6) | 8.4 (7.3,13.9) | 0.6 (-1.8,6.6) | 0.36 |
| Front-back | 2.9 (2.1,3.3) | 2.5 (1.6,3.7) | -0.4 (-1.2,0.7) | 0.29 | 2.8 (2.1,3) | 2.9 (2.6,3.6) | 0.1 (-0.7,1) | 0.29 |
| Hand-jersey | 10.7 (8.6,14.4) | 9.5 (8,11.7) | -1.2 (-3.6,0.9) | 0.21 | 9.6 (9.2,10.2) | 13 (8.9,16.4) | 3.4 (-1.4,7.4) | 0.13 |
| Hand-hand | 1 (0.7,1.7) | 1.9 (1.3,2.4) | 0.9 (0.3,1.5) | 0.008 | 1.9 (1.3,2.8) | 0.7 (0.6,1) | -1.2 (-2.4,-0.4) | 0.007 |
| Hugging | 0 | 0 (0,0.1) | 0 | 0.76 | 0.1 (0.1,0.2) | 0 | -0.1 (-0.2,0) | 0.02 |
| Head contacts | 0.3 (0.2,0.4) | 0.3 (0,0.7) | 0.1 (-0.2,0.4) | 0.83 | 0.3 (0.3,0.7) | 0.3 (0.2,0.4) | -0.1 (-0.4,0.1) | 0.12 |
| Head-head | 0 | 0 | 0 (-0.1,0.1) | 0.43 | 0 (0,0.1) | 0.1 (0,0.1) | 0 (0,0.1) | 0.96 |
| Arm-head | 0.3 (0.1,0.4) | 0.3 (0.1,0.7) | 0 (-0.3,0.3) | 0.88 | 0.4 (0.2,0.6) | 0.3 (0.1,0.4) | -0.1 (-0.4,0.1) | 0.12 |

**TABLE S2** Number of involved players and duration of crowding during breaks. Data are presented as frequency per player-hour and as median with interquartile range. Differences between matches played in spring vs. fall and before and after the lockdown are displayed as median differences with 95% confidence intervals. P values from Kruskal-Wallis test.

|  | **spring** | **fall** | **Δ** | **P value** | **pre lockdown** | **post lockdown** | **Δ** | **P value** |
| --- | --- | --- | --- | --- | --- | --- | --- | --- |
| Free-kick wall |  |  |  |  |  |  |  |  |
| N players | 2.5 (2,2.8) | 2.5 (2,3.1) | 0 (0,0) | 0.52 | 2.4 (2,2.7) | 2 (0.5,3.3) | -0.4 (-2.7,0.6) | 0.51 |
| duration [s] | 15 (8.5,21.7) | 15.2 (9.3,21.5) | 0.2 (-6,6.2) | 0.91 | 21.9 (15.6,25.6) | 19.4 (3.5,24.3) | -2.4 (-20.9,7.3) | 0.41 |
| Goal celebration |  |  |  |  |  |  |  |  |
| N players | 3.9 (0,5.4) | 5.6 (0,8) | 1.7 (-3.8,6.4) | 0.07 | 6.2 (5.3,7.7) | 1.9 (0,4.4) | -4.3 (-7.4,-1.3) | 0.004 |
| duration [s] | 4 (0,8.9) | 5.4 (0,9.8) | 1.4 (-4.3,5.9) | 0.38 | 9.8 (4.4,15.3) | 1.5 (0,5.5) | -8.3 (-15,-0.5) | 0.02 |
| Corner / free-kick |  |  |  |  |  |  |  |  |
| N players | 5.7 (4.7,6.5) | 5.6 (4.3,6.5) | -0.1 (-1.2,1.1) | 0.95 | 6 (5.4,7.5) | 6.3 (5.9,7.3) | 0.3 (-2.1,1.6) | 0.55 |
| duration [s] | 6.2 (5.5,7.6) | 6.9 (5,8.9) | 0.8 (-1.2,2.7) | 0.47 | 7.8 (5.9,8.2) | 6.5 (5.9,7.3) | -1.3 (-3.8,0.5) | 0.38 |
| Injury |  |  |  |  |  |  |  |  |
| N players | 4.9 (3.9,6) | 4.3 (0,5.5) | -0.6 (-2.8,0.7) | 0.21 | 4.2 (1,5.2) | 4.3 (3.6,4.7) | 0.1 (-1.4,4.3) | 0.94 |
| duration [s] | 10 (6.7,15.8) | 7.1 (0,13.5) | -2.9 (-8,4.1) | 0.16 | 8.5 (1.4,11.1) | 13.7 (8.9,15.8) | 5.2 (-1.8,13.7) | 0.10 |

**TABLE S3** Actions of individual players representing potential risk situations associated with the transmission of respiratory diseases (by aerosol or droplet production) and within-player hand-to-head contacts. Data are presented as frequency per player-hour and as median with interquartile range. Differences between matches played in spring vs. fall and before and after the lockdown are displayed as median differences with 95% confidence intervals. P values from Kruskal-Wallis test.

|  | **spring** | **fall** | **Δ** | **P value** | **pre lockdown** | **post lockdown** | **Δ** | **P value** |
| --- | --- | --- | --- | --- | --- | --- | --- | --- |
| Aerosol or droplet production | 2.5 (1.7,3.8) | 2.8 (1.1,4.4) | 0.4 (-0.7,1.3) | 0.49 | 3.8 (2.8,5.1) | 2.5 (2.1,3.7) | -1.3 (-2.9,0.4) | 0.02 |
| Speaking | 1.8 (1.1,2.8) | 2.4 (1,3.1) | 0.6 (-0.4,1.4) | 0.49 | 2.9 (1.9,3.9) | 1.7 (1.4,2.5) | -1.2 (-2.4,0.1) | 0.03 |
| Shouting | 0.1 (0,0.3) | 0.1 (0,0.2) | 0 (-0.2,0.1) | 0.65 | 0.3 (0.2,0.4) | 0.2 (0.1,0.2) | -0.1 (-0.2,0) | 0.11 |
| Spitting | 0.5 (0.2,0.7) | 0.2 (0,0.8) | -0.2 (-0.5,0.3) | 0.28 | 0.7 (0.5,0.8) | 0.7 (0.6,0.8) | 0 (-0.3,0.3) | 1.00 |
| Hand-to-head | 21.6 (16.7,30.5) | 23 (17.7,27,2) | 0.9 (-6.9,6.9) | 0.88 | 16.4 (14.5,19.1) | 16.8 (15.8,18.2) | 0.4 (-2.9,4.5) | 0.60 |
| mucosal | 12.4 (10.4,16.9) | 12.6 (9.7,15.5) | -0.3 (-3.9,2.6) | 0.43 | 10.9 (10.4,11.4) | 10.9 (10.4,11.4) | 0 (-1.6,2.8) | 0.65 |
| non-mucosal | 9.4 (6,16.1) | 9.6 (7.8,13.2) | 0.2 (-3.9,3.9) | 0.77 | 5.8 (4.5,6.6) | 6.2 (4.9,6.5) | 0.4 (-1.2,2.0) | 0.76 |

**TABLE S4** Hand (or head)-to-ball contacts during match play and set-play situations, including attempts to place the ball. Data are presented as frequency per player-hour and as median with interquartile range. Differences between matches played in spring vs. fall and before and after the lockdown are displayed as median differences with 95% confidence intervals. P values from Kruskal-Wallis test.

|  | **spring** | **fall** | **Δ** | **P value** | **pre lockdown** | **post lockdown** | **Δ** | **P value** |
| --- | --- | --- | --- | --- | --- | --- | --- | --- |
| Throw-in | 37 (28.8,46) | 39 (32.6,51.2) | 2 (-7.7,12.7) | 0.34 | 28.7 (27.3,30.8) | 29.3 (27,34.8) | 0.7 (-5.7,6.3) | 0.60 |
| Corner | 6.7 (4,13.2) | 8 (5.8,11.3) | 1.3 (1.3,1.3) | 0.49 | 4.7 (4,7.3) | 4.7 (2.8,6.7) | 0 (-3.7,2.7) | 0.60 |
| Free-kick | 18.3 (14.2,21.8) | 20 (6.8,22.5) | 1.7 (-10.8,6) | 0.48 | 20 (18,22.8) | 19.7 (16,21.2) | -0.3 (-4.7,3.3) | 0.57 |
| Goal kick | 12.7 (9.3,19) | 15.1 (12,21) | 2.5 (-1.3,7.2) | 0.08 | 11.3 (10.7,12) | 9.3 (7.5,11.2) | -2 (-4.7,0) | 0.12 |
| Kick-off | 2 (0.2,3.6) | 1.3 (0.9,3.6) | -0.7 (-0.7,-0.7) | 0.07 | 1.3 (0.7,1.3) | 0.3 (0,1.3) | -1 (-2,0) | 0.34 |
| Drop ball | 0.7 (0,1) | 0 (0,0.7) | -0.7 (-0.7,-0.7) | 0.13 | 0.7 (0.2,0.7) | 0.3 (0,0.7) | -0.3 (-0.7,0) | 0.45 |
| Kick-out | 13 (9.3,23.8) | 16.7 (11,25.1) | 3.7 (-5.7,12.7) | 0.42 | 9.7 (8,11.8) | 9.7 (8.3,12.2) | 0 (-3.3,3) | 0.62 |
| Goalkeeper save | 4 (2.7,7) | 5.5 (3.7,10.9) | 1.5 (-1.7,5.9) | 0.32 | 1.7 (0.8,4) | 3 (2.7,4) | 1.3 (-2,2.7) | 0.19 |
| Header | 58.8 (48,72.8) | 59.5 (31.3,79.5) | 0.7 (-26.5,21) | 0.64 | 62.7 (46.3,77.2) | 59 (51.7,78.8) | -3.7 (-28,18.3) | 0.97 |

**TABLE S5** Hand (or head)-to-ball contacts during match play and set-play situations, including attempts to place the ball. Data for the different playing levels are presented as frequency per match-hour and as median with interquartile range. Differences between playing levels are displayed as medians with 95% confidence intervals. P values from Kruskal-Wallis test.

|  | **all matches** | **professional** | **amateur** | **youth** | **Δ youth – professional** | **Δ amateur – professional** | **Δ amateur – youth** | **P value** |
| --- | --- | --- | --- | --- | --- | --- | --- | --- |
| Throw-in | 38 (29.5,47) | 28.7 (27.2,32) | 38 (34.2,39.7) | 49.6 (45.8,63.1) | 20.9 (16.2,32.2) | 9.3 (4,13.7) | -11.6 (-21.6,-5) | < 0.001 |
| Corner | 7.7 (4.7,11.8) | 4.7 (3.3,6.8) | 6.7 (5,8) | 13 (10,15.9) | 8.3 (5.2,11.1) | 2 (-1.3,4) | -6.3 (-9.7,-3.3) | < 0.001 |
| Free-kick | 19 (10.2,22) | 20 (17.3,21.8) | 24 (22,25.3) | 7.6 (6,12.3) | -12.4 (-15,-9.2) | 4 (4,4) | 16.4 (13,19.2) | < 0.001 |
| Goal kick | 13.7 (10.7,20.9) | 10.7 (9,12) | 13.3 (10.2,14) | 22 (19.2,27) | 11.3 (7.4,14.7) | 2.7 (-2.7,4) | -8.7 (-13.4,-6) | < 0.001 |
| Kick-off | 1.7 (0.7,3.6) | 1 (0,1.3) | 1.3 (1.3,2.5) | 4.3 (2,6.3) | 3.3 (1.3,5.3) | 0.3 (-1.3,1) | -3 (-5.2,-0.9) | < 0.001 |
| Drop ball | 0.3 (0,0.9) | 0.7 (0,0.7) | 0.3 (0,1.2) | 0 (0,1) | -0.7 (-0.7,-0.7) | -0.3 (-0.7,0.3) | 0.3 (-1,1.3) | 0.93 |
| Kick-out | 13.7 (9.5,24.8) | 9.7 (8,12) | 12.3 (10.3,17.3) | 25.1 (20,32.7) | 15.4 (11.4,22.1) | 2.7 (-0.3,9.3) | -12.8 (-20.4,-6.3) | < 0.001 |
| Goalkeeper save | 4.7 (2.7,8.9) | 2.7 (1.3,4) | 4 (3,5.7) | 10.2 (7.2,12.3) | 7.5 (5,9.3) | 1.3 (-1.3,2) | -6.2 (-8.8,-4) | < 0.001 |
| Header | 59 (41.5,78.9) | 59.7 (49.3,79.5) | 80.7 (78,90.7) | 42.3 (25.2,55.3) | -17.4 (-35.7,0.3) | 21 (1.3,33.3) | 38.4 (18.8,54.7) | < 0.001 |
